# Periodic Fever, Aphthous Stomatitis, Pharyngitis and Adenitis Syndrome: An Update

**DOI:** 10.3390/children12040446

**Published:** 2025-03-30

**Authors:** Maria Michailou, Chryssoula Perdikogianni

**Affiliations:** Department of Pediatrics, Medical School University of Crete, 70013 Heraklion, Greece

**Keywords:** PFAPA syndrome, children, adolescents, genetics, autoinflammatory diseases

## Abstract

Periodic Fever, Aphthous Stomatitis, Pharyngitis and Adenitis (PFAPA) syndrome is an autoinflammatory disorder of unknown genetic etiology typically characterized by recurrent fever episodes, pharyngitis, aphthous stomatitis and cervical lymphadenitis. The syndrome runs a benign course with fever episodes recurring in regular intervals and usually resolves around adolescence, even though, for a subset of patients, it persists through adulthood. It is considered a condition of multifactorial etiology, a syndrome that is triggered by environmental factors in genetically predisposed individuals. Increasing evidence points towards a correlation between monogenic autoinflammatory syndromes and PFAPA syndrome both in pathogenesis and therapeutic approaches. In this review, we present an update of the current literature on PFAPA and focus on new data on genetics of PFAPA and the association of PFAPA with other autoinflammatory diseases.

## 1. Introduction

Periodic Fever, Aphthous Stomatitis, Pharyngitis and Adenitis (PFAPA) Syndrome is an autoinflammatory fever syndrome of unknown etiology, characterized by recurrent fever episodes with regular intervals between episodes along with pharyngitis, aphthous stomatitis and cervical lymphadenitis. It was first described by Marshall et al. in 1987 with a set of criteria that has since been revised by Thomas et al. [1]. The syndrome’s cumulative incidence is 2.3 cases in 10,000 children (under 5 years old) with a slight male predominance in childhood [1,2,3]. Disease onset is usually before the age of 5 years, and it resolves during adolescence, even though adult onset PFAPA cases have been described in the literature [1]. PFAPA febrile episodes last 4–5 days, are accompanied by pharyngitis, lymphadenitis or aphthous stomatitis, and recur at regular intervals (2–8 weeks). The patients’ growth is not affected, and diagnosis of the syndrome relies on the exclusion of cyclic neutropenia, upper respiratory infections or other periodic fever syndromes [1,3,4].

Several theories have been proposed with regard to the causes of the syndrome, with the predominant one being immune dysregulation in genetically predisposed individuals, resulting in an exaggerated response to environmental triggers [1]. Several studies have indicated alterations in the immunologic profile of the patients during the episodes with dysregulation of the innate immune system, as well as anatomical and biochemical differences in the tonsillar tissue of the patients [1,5,6]. The lack of positive microbial cultures, response to antibiotics, exposure to infected individuals, and the cessation of symptoms after corticosteroid administration disprove the theories of infectious origin of the syndrome.

PFAPA syndrome had been considered a non-hereditary autoinflammatory disease despite the fact that the existence of family pedigrees might suggest an autosomal dominant pattern of inheritance with variable penetration [7]. The high prevalence of heterozygotes of monogenic autoinflammatory diseases, such as Familial Mediterranean Fever (FMF), mevalonate kinase deficiency (MVK) or Cryopyrin-Associated Periodic Syndrome (CAPS), among PFAPA patients challenged that notion and those genes are now considered disease modifiers. Currently, PFAPA tends to be considered a syndrome of oligogenic or multigenic etiology [3,8,9,10].

The management of PFAPA has two targets: the control of acute attacks and a decrease in episode frequency. Non-Steroidal Anti-inflammatory Drugs (NSAIDs), corticosteroids and, recently, IL-1 inhibitors have all shown variable degrees of efficacy in the cessation of the episodes, while colchicine, cimetidine and anakinra have been shown to increase the intervals between febrile episodes [1,2,8]. The use of vitamin D, pidotimob [11] and probiotic K12 has been proposed in certain studies but needs further research to credit it.

## 2. Methodology

The terms “PFAPA”, PFAPA AND GENETICS”, “PFAPA AND BEHCET”, “PFAPA AND FMF” were searched in the title and abstract in the Pubmed search engine and in the sections of review, systematic review, meta-analysis, clinical trial and randomized clinical trials, and we opted for articles published within the last 10 years in the English language. Of the articles that emerged, the most recent ones were selected, and especially the ones that focused on pathogenesis, genetics and treatment outcome.

## 3. Pathophysiology

The etiopathogenesis of PFAPA is largely unknown. All clues point to a multifactorial disease. The infectious origin of the disease has been discredited due to the absence of positive microbial cultures or serological findings, a lack of response to antibiotics, and the cessation of fever after corticosteroid therapy. The differences in the tonsillar microbiome of children with PFAPA (higher prevalence of Candida Albicans, lower viral load) are mostly attributed to children’s increased exposure to antibiotics prior to the diagnosis [1,6,12,13]. The hypothesis that the use of probiotic Streptococcus K12 could influence the course of the disease as it decreases the frequency of pharyngotonsillitis and acute otitis media has been tested by La Torre et al. In their study, the administration of this probiotic for a period of 6 months appeared to reduce the frequency and the duration of the flares. Furthermore, the discontinuation of the treatment reversed these benefits [14]. PFAPA syndrome is currently largely considered the result of immune system dysregulation and several facts point towards this assumption. PFAPA attacks are associated with a notable increase in inflammatory proteins (CRP, ESR, serum amyloid protein SAA), lymphopenia and neutrophil dysfunction (neutrophil apoptosis, priming and generation of oxidative burst) [1], which subside in the asymptomatic intervals. IL1-β overexpression has been associated with the syndrome’s pathogenesis and is regulated by inflammasomes that control the activation of caspase-1 (protein that cleaves pro IL-1β and pro IL-18, therefore increasing IL-1 concentration in the plasma) [15].

PFAPA’s high resolution rates after tonsillectomy have suggested a primary role of the tonsils in the pathogenesis of the syndrome. Dytrych et al. compared the tonsil tissue of children with PFAFA and children with obstructive apnea. They found differences both in the tonsillar white blood cell populations (lower percentage of B lymphocytes CD 19+ and a proportional increase in CD8+ T cells) and the chemokine profile (CXCL20, CXCL9, CCL19 genes significantly higher in children with PFAPA) [6]. Tonsillar germinal centers were also found to be smaller with a wider average squamous epithelium in PFAPA patients, and a different pattern of localization of antimicrobial peptides (human β defensin) was noted in patients. Ultimately, a shift in the expression of chemokines in the tissue (lower gene expression of IL-4 compared to controls, with similar expression of IL1-β, IL-17, TNF-a, TGF-β) points towards an inhibition of Th2 expression, which could be linked to the syndrome’s pathogenesis [4,5]. Larger germinal cells and lower interleukin-1 receptor antagonist (IL 1RN) expression have been associated with longer intervals between episodes [16]. The genetic component of PFAPA pathogenesis has been a point of dispute as, despite the syndrome’s strong familial clustering [7,17,18], it cannot be attributed to monogenic mutations and has been classified as a non-hereditary autoinflammatory disorder [19,20]. Cheung et al. demonstrated associations between monogenic autoinflammatory disorders genes and PFAPA disease and discovered a variant in the CARD8 gene that was more common in PFAPA patients compared to the controls (14% vs. 3.2%). Individuals with PFAPA heterozygous for mutations in the MEFV (FMF), NLRP3 (CAPS), MVK (MKD/HIDS), TNFRSF1A (TNF-receptor associated periodic syndrome -TRAPS), and CARD15/NOD2 (Blau syndrome) genes often exhibit atypical symptoms like abdominal pain and arthralgias. These genes seem to act as disease modifiers for PFAPA and affect the response to pharmacological or surgical therapy [21]. Additionally, common alleles of the gene NOD2 (R702W, G908R, and 1007fsinC), associated with Crohn’s disease, were found in children with PFAPA and conferred a PFAPA phenotype with abdominal symptoms [3]. Recently, ALPK1 kinase, a protein associated with the function of NLRP3 inflammasome, has been associated with the febrile manifestations of PFAPA syndrome [22]. Epigenetic changes such as differences in methylation of Phosphoinositide-3-Kinase Adaptor Protein 1 (PIK3AP1) and spondin 2 (SPON2) gene could also be associated with the pathogenesis of PFAPA syndrome [23].

There are indications that oxidative stress and metabolic dysregulation could be implicated in the pathogenesis of PFAPA syndrome. Reactive oxygen species and antioxidant mechanisms are balanced in healthy individuals and are key components in homeostasis. Increased oxidative stress causes damages in proteins, lipids and DNA molecules. Tuğrul et al. showed increased oxidative stress and DNA damage in PFAPA patients [24].

Vitamin D regulates immunity by interacting with receptors to modulate T cell proliferation and dendritic cell function. Vitamin D deficiency has also been found in high prevalence in autoimmune diseases such as rheumatoid arthritis, systemic sclerosis and Systemic Lupus Erythematosus (SLE). Several studies have associated Vitamin D deficiency in PFAPA patients with higher inflammatory markers and duration of the disease [25,26,27,28].

## 4. Clinical Manifestations and Epidemiology

PFAPA usually presents with fatigue as a prodomal symptom. The fever flares last 2–8 days (mean duration 5 days) and recur every 2–8 weeks. During febrile episodes, children often show erythematous or exudative pharyngitis (90%), cervical lymphadenitis (>75%), and oral aphthous lesions in 50% of cases [4]. Abdominal pain, arthralgia, arthritis, headache and rashes can also accompany a PFAPA fever attack. Rhinorrhea and cough are absent from PFAPA flares, as they are mostly indicative of upper respiratory infections. There have been several cases of adult onset PFAPA described in the literature and in this age group joint symptoms, along which myalgia and headache are more common.

PFAPA is the most common fever syndrome in non-Mediterranean countries. It has an estimated prevalence of 2.3/10,000 in Nordic Countries (2.3/10,000 in Norway [29,30], 2/10,000 in Finland in children under 5 years [31]). Ng et al. reported an incidence rate of 0.098/10,000 in children aged 0–16 years in an English tertiary hospital, with a rise of 0.247/10,000 during the COVID-19 pandemic [32]. Male predominance is documented amongst the affected children (53–72%) [1,2,3]. The use of international registries could probably shed light into the epidemiology and incidence of the syndrome [33].

## 5. Diagnosis

Marshall et al. first described the syndrome in 1987 and came up with a set of criteria that has since been revised by Thomas in 1999. This set of criteria diagnoses PFAPA patients with great sensitivity, but lacks specificity, especially when it comes to distinguishing PFAPA and FMF or other monogenic autoinflammatory syndromes [1,6,34,35]. The estimated specificity of the Marshall–Thomas criteria depends vastly on the FMF prevalence of the investigated population, varying from 38 to 61% [34]. Several attempts have been made to increase the specificity of the PFAPA diagnosis and to include a wider range of patients. Cantarini et al. published a new set of criteria to include late-onset PFAPA patients, who exhibit an atypical presentation with pronounced fatigue, arthralgias and myalgias, without exudative tonsillitis (Table 1) [35,36]. Vanoni et al.’s criteria proved to be too restrictive and reduced the sensitivity [37,38]. Takeuchi et al. proposed a new set of criteria in 2019 with a reported high sensitivity and specificity that adds family history, response to corticosteroids and high IgD to the diagnostic process [39].

Eurofever criteria for PFAPA are of limited use in clinical diagnosis but serve as classification criteria in clinical studies and help to distinguish autoinflammatory diseases with similar manifestations [40].

Fever diary is an important tool to prove the periodicity of the syndrome. Blood tests during PFAPA flares show a significant increase in inflammatory markers such as CRP and serum Amyloid A (SAA) and should be repeated on the seventh to fourteenth day of the flare to prove the fall of these markers [3]. PFAPA patients exhibit normal growth and are completely asymptomatic between flares. It is also mandatory to exclude other conditions such as upper respiratory infections, cyclic neutropenia, immunodeficiency and neoplastic diseases. Several biomarkers have been proposed through the years, but are currently not in use in daily clinical practice [39,41].

## 6. Differential Diagnosis

PFAPA syndrome is an autoinflammatory disorder with systematic symptoms that closely resemble other conditions (Table 2) [19,42,43].

Cyclic neutropenia is a disease characterized by periods of severe neutropenia (neutrophils under 500) that occur with 21-day intervals. As a result, patients during the neutropenic phase suffer from febrile attacks and oral aphthous ulcers, gingivitis and bacterial infections. The attacks do not respond to single-dose corticosteroid therapy and the diagnosis is made through genetic tests (ELANE gene mutations) [1,3]. The exclusion of upper respiratory infections is also included in the diagnostic criteria of PFAPA. Upper respiratory infections happen more often during the winter months, lack periodicity, do not respond to corticosteroids and may respond to antibiotics [9]. Monogenic Autoinflammatory disorders such as MKD, TRAPS, CAPS, and FMF present in a similar manner to PFAPA and the Gaslini score is used to distinguish the group of patients that would benefit from further genetic investigation (Table 3). The score values the age of onset, presence of abdominal pain, chest pain and diarrhea. Exudative tonsillitis is present in PFAPA and not in monogenic autoinflammatory diseases. Skin rashes, arthritis, diarrhea, chest pain, hepatosplenomegaly, fever episodes longer than 7 days, a history of hearing loss or episodes associated with cold or exercise are symptoms that are not supportive of PFAPA diagnosis [3,9].

In FMF, fever episodes last 12–72 h, and are usually associated with severe abdominal pain, pleurisy or arthritis, erysipelas like rash and orchitis [3]. MKD is a rare autosomal recessive condition that presents like PFAPA with recurrent fever, oral aphthous lesions and cervical adenopathy, but is also accompanied by abdominal pain, diarrhea, vomiting, skin rash, generalized lymphadenopathy, splenomegaly, arthralgia, arthritis or myalgia. The episodes are triggered by immunization at an early age [9]. TRAPS involves irregular, prolonged fevers, migratory skin rash, periorbital swelling, conjunctivitis, pleurisy, and lacks vomiting and aphthous stomatitis [3]. Behcet disease is a chronic inflammatory disease presenting with ulcerative disease (oral and/or genital), gastrointestinal symptoms, neurological symptoms and vasculitis [44].

## 7. Associations Between PFAPA and FMF

More than 30 monogenic autoinflammatory syndromes have been recognized, the most common of which in the Mediterranean area is Familial Mediterranean Fever. PFAPA, on the other hand, is considered an oligogenic disease nowadays, the pathogenesis of which could be lying in the IL-1β pathway. FMF is the result of defective pyrin, a protein that regulates apoptosis and inflammation. The over 80 recognized mutations of the MEFV gene result in overactivation of the IL-1β pathway. Therefore, the question is whether these two clinical entities could have a genetic overlap [9,34,45].

Most studies confirm a prevalence of MEFV mutations in PFAPA patients, ranging from 27 to 65% [19,43,46,47,48,49]. Heterozygotes of MEFV mutations in PFAPA patients present a slightly more favorable phenotype as they appear to experience shorter PFAPA flares, decreased frequency of oral ulcers, and a positive response to lower corticosteroid dosage and to colchicine. MEFV gene carriers also display lower remission rates after tonsillectomy [46,50]. Heterozygotes of MEFV are also represented with a higher frequency in other inflammatory disorders such as Behcet’s disease, Crohn’s disease, ulcerative colitis and Henoch Schoenlein Purpura [51]. FMF carriers also have lower rates of asthma than the general population. One of the circulating hypotheses is that FMF gene carriers are more prone to Th1 over Th2 system activation, therefore being in a constant “proinflammatory state”, making them susceptible to external triggers and the development of PFAPA syndrome [50].

Recent research points towards an oligogenic pattern of inheritance in PFAPA patients depending on the “total inflammatory burden”. The accumulation of multiple inflammasome-associated variants affects the severity of the phenotype and the response to therapy happens in a dose-dependent manner. Federici et al. proposed that this “dose-effect” was associated with MEFV mutations as well, as individuals carrying low penetrance MEFV mutations were more likely to experience more PFAPA like symptoms and less FMF like symptoms compared to individuals carrying two more high penetrance FMF mutations [50]. On the other hand, several facts appear to disprove this association. Whole blood gene profiling points towards differences in gene expression between PFAPA and FMF patients, involving at least 600 different genes related to cytotoxic T lymphocyte-related apoptosis of target cells, T cell receptor signaling, IL-9 signaling, p53 signaling, and TREM1-signaling pathways. Transcription patterns during PFAPA flares suggest involvement of host defense mechanisms comprising innate and adaptive immunity, supporting the theory of immunologic response to environmental trigger. None of the genes involved in monogenetic autoinflammatory syndromes could be associated with similar molecular biology [9].

## 8. Associations Between PFAPA and Behcet Disease

A possible link between PFAPA and Behcet disease has recently emerged in the literature. These two entities present with episodic febrile attacks with oral ulcers. The similarity in pathogenesis is attributed to imbalance of the IL-1β pathway and over-activation of Th1-mediated immunity [52]. Cantarini et al. found a relatively high percentage (7.5–30%) of Behcet patients that fulfilled the criteria for PFAPA in their childhood [53,54]. Bedir et al., in their case–control study, found a high frequency of HLA-B15 in both diseases [55]. Manthiram et al. demonstrated the existence of common pathogenic loci (IL-12A, IL-10, STAT4, CCR1-CCR3) [56]. The genetic correlation is emphasized by the observation that 5 % of PFAPA patients have a relative with Behcet [55].

Manthiram et al. expressed the theory that these two diseases could represent a continuum of oral ulcers associated diseases, with Behcet being the most severe condition of the spectrum, PFAPA an intermediate condition, and recurrent oral aphthosis (RAS) representing a milder condition. Researchers hypothesize that the difference in Major Histocompatibility Complex (MHC) observed amongst these patients could influence the clinical phenotype, thus resulting in the heterogeny of the disease [44,56]. Despite the convincing arguments, there are some elements contradicting this theory. PFAPA and Behcet disease have different ages of onset (childhood vs. adulthood) and the clinical presentation differs as PFAPA patients have tonsillitis and lymphadenopathy, while Behcet patients have ocular redness, uveitis and testicular pain. PFAPA is usually associated with a more pronounced increase in inflammatory markers [55]. Behcet disease distinguishes itself from other autoinflammatory diseases as it combines Th1- and Th2-mediated responses, unlike PFAPA, which is mostly considered a Th1 disease [53].

## 9. Management of PFAPA

The two main goals of PFAPA management are to control acute attacks and decrease attack frequency or even prevent future attacks (Table 4 and Table 5) [2,37,57,58].

The therapeutic measures for the treatment of acute attacks that have been suggested in the literature through the years are NSAIDs (as a supportive measure), and corticosteroids, such as prednisolone in a dose of 1–2 mg/kg (has been proven to cause the cessation of the attack, even though systematic use could decrease the interval between the attacks) [2,28,37].

The prevention of future episodes or an increase in the interval between febrile attacks could be achieved through several medical regimens such as cimetidine [2] (debatable efficacy, ranges between 27 and 42% in studies), colchicine [59] (increased flare interval from 20 to 50 days in 85% of patients), IL-1 inhibitors (limited use due to increased cost of the therapy, more painful injections) [2,28,37], vitamin D (lower levels of vitamin D have been associated with higher inflammatory markers and more frequent episodes), pidotimob, ketotifen [60], and probiotic K12 (decreases the frequency of tonsillitis, debatable efficacy in PFAPA syndrome). There are even some anecdotal reports supporting the efficacy of lidomide and montelukast in PFAPA patients. There have also been some studies suggesting the use of thalidomide in adult PFAPA patients, as it is associated with a reduction in oral ulcers and systemic symptoms [11].

Tonsillectomy is considered an effective therapy, as a meta-analysis reports an efficacy of 64–100% with a complete remission in a large number of the cases [58,61,62]. However, a limited number of patients will be candidates for surgery due to the known self-limited course of the disease and the high rates of success of drug therapy. Heterozygotes of the MEFV genes display lower rates of PFAPA remission after tonsillectomy [2,50,61,62,63,64]. Glucocorticoid therapy (prednisolone 1–2 mg/kg up to 25–60 mg, or betamethasone 0.1–0.2 mg/kg/dose) consists of the administration of a single dose early during the fever flare. More than 90% of patients will experience absence of the fever within a few hours, but some fatigue may persist for several hours or days. Yazgan et al. showed that lower doses of corticosteroids could achieve similar results (mean dose 0.59 mg/kg/dose) even though these slightly increased the hours needed for the cessation of fever (6–8 h in higher doses vs. 8–12 h) [2]. The exact mechanism of action of colchicine in autoinflammatory syndromes is still unknown. Colchicine prevents the polymerization of microtubules, which are key components of the cytoskeleton, and results in RhoA kinase activation. The activation of this biochemical path results in the suppression of pyrin inflammasome, therefore limiting the production of IL-1β [50]. The prevalence of MEFV heterozygotes within PFAPA patients suggests that the control of disease flares could be achieved in a similar manner in selective cases [4,5,65,66]. Butbul et al., in their randomized trial with 18 children, showed that colchicine therapy could reduce attack frequency, but larger randomized studies need to be conducted to prove its efficacy [58,67,68]. Raeskarami et al., in their randomized controlled trial of 67 PFAPA patients, showed that there is no statistically significant difference between colchicine and cimetidine as a prophylactic measure [69].

IL-1β inhibitors such as Anakinra have been recently used as alternatives to the previous regimens both as flare suppressants and long-term treatment for a reduction in the episodes (interval between attacks and number of attacks). The use of IL-1β inhibitors has been limited due to cost effectiveness (expensive therapy in a self-limited syndrome), difficulties in administration (painful subcutaneous injections), possibility of immunosuppression, and the parents’ resistance to consent to the administration of the therapy [1].

Pidotimob (3-L-pyroglutamyl-Lthiazolidine-4carboxylic acid) is an immunomodulatory agent that has been shown to increase antigen presentation and promote adaptive Th-1-mediated immunity, as well as increase the concentration of salivary IgA. Buongorno et al. showed that the administration of the molecule led to a 68% remission rate for the participants of the study (37 children). Similarly, Manti et al. showed that 22 patients that received the therapy showed a significant reduction in the frequency of febrile episodes, pharyngitis and aphthous stomatitis [11]. Further investigations are needed in order to evaluate the possibility of wider use of this therapeutic option.

Due to the benign nature of the disease and the limited epidemiological data, there was a lack of evidence-based evaluation of therapeutic plans in clinical trials. The Eurofever and the AIDA registry offered important clues and enabled the evaluation of the most commonly used therapies, such as corticosteroids, colchicine, cimetidine and tonsillectomy [33,58]. The Childhood Arthritis and Rheumatology Association (CARRA) issued consensus treatment plans in 2020 which mostly focused on defining corticosteroid-responsive cases and divided the approach into antipyretic, corticosteroid, prophylaxis and surgical therapy (Table 6) [70].

## 10. Disease Course and Prognosis

PFAPA syndrome usually runs a benign course, associated with spontaneous resolution within 3–5 years of disease onset. Wurster et al. demonstrated that 85% of patients experienced complete remission within 6 years, although 15% of patients remained symptomatic for a mean period of 18 years [1,3]. A later age of onset, a positive family history of PFAPA syndrome, and the absence of headaches are considered independent prognostic factors, indicating the likelihood of resolution of the syndrome within 4 years of onset [67]. A French study found that PFAPA patients experience reduced quality of life, with increased fatigue and significant impacts on psychosocial functioning, as measured by the Pediatric Quality of Life Inventory score [2]. Similarly, Bedir et al., in a recent study, showed a significant reduction in quality of life in sixty PFAPA and thirty Behcet patients compared to a control group using the same score [55].

## 11. PFAPA and Undifferentiated Recurrent Fever Syndrome (SURF)

Hausmann et al. recognized a subset of patients that present with recurrent or periodic fever but do not fit the diagnostic criteria for PFAPA and do not have any known genes for monogenic autoinflammatory diseases. Especially in the pediatric population, these patients tend to be mislabeled as PFAPA. They named this entity “syndrome of undifferentiated recurrent fevers” (SURF) [35].

Luu et al. recognized that SURF patients tend to have gastrointestinal symptoms more frequently, are less likely to respond to corticosteroids compared to PFAPA patients and experience remission after tonsillectomy. Furthermore, on pathological examination of the tonsillar tissue, they presented reduced numbers of CD3+ cells compared to PFAPA patients and a strong IL-1 expression, similarly to PFAPA patients [71].

Papa et al. recommended a set of features that would point towards the classification of these patients under this term, with mandatory indications being recurrent fever with high inflammatory markers, not fulfilling the PFAPA criteria, and negative genetic testing for monogenic autoinflammatory diseases [72]. Broderic et al. suggest a diagnostic algorithm to guide the clinician in the diagnosis of recurrent fevers [73].

Further research needs to be carried out to clarify whether SURF constitutes a separate entity with a common pathogenetic mechanism or whether it represents a subcategory of atypical PFAPA presentations.

## 12. Conclusions

The present literature review attempts to examine the possible correlation of PFAPA syndrome with monogenic autoinflammatory syndromes and Behcet disease, as well as the implications these findings could have on the treatment of these patients. This review was not designed to be a systematic analysis of the current data, and this can be considered a limitation.

The benign course of the disease has hindered timely diagnosis and recording of these patients. More randomized controlled trials are needed to evaluate the potency of both the established and experimental therapies. PFAPA is a self-limited condition with multifactorial origin that does not affect the patients’ growth or long-term quality of life. The overlap of the clinical symptoms with monogenic autoinflammatory syndromes such as Familial Mediterranean Fever demands physicians’ attention as the co-existence of genes associated with monogenic autoinflammatory diseases could alter the clinical presentation and therapeutic management. More research needs to be carried out to clarify both the pathogenesis and the management options of the syndrome.

## Figures and Tables

**Table 1:** Modified Marshall criteria and Cantarini criteria for adult patients.

Criteria	Modified Marshall Criteria	Cantarini Criteria
Age of Onset	Childhood	At least 16 years old
Main Symptoms	Recurrent fever with general symptoms, absence of upper respiratory infection signs	Recurrent fever
Additional Symptoms	At least one of the following: aphthous stomatitis, cervical lymphadenopathy, pharyngitis	Erythematous pharyngitis during fever and/or cervical lymphadenitis during fever
Inflammatory Markers	Not specified	Increased during attacks
Symptom-free Intervals	Yes	Yes
Exclusion Criteria	Cyclic neutropenia	Infective, autoimmune, neoplastic diseases, monogenic and polygenic autoinflammatory diseases
Throat Swab	Not specified	Negative during fever
Antibiotic Therapy	Not specified	Ineffective

**Table 2:** Differential diagnosis of PFAPA syndrome.

Differential Diagnosis of PFAPA Syndrome		
Disease	Symptoms	Notes
Cyclic neutropenia	Severe neutropenia with 21-day intervals, fever, oral aphthous ulcers, gingivitis, bacterial infections	ELANE gene mutations
Behcet’s disease	Ulcerative disease (oral and genital), gastrointestinal symptoms, neurological symptoms, vasculitis	Clinical criteria, multigenetic disease
Monogenetic Autoinflammatory Diseases		
FMF	Fever episodes (12–72 h), severe abdominal pain, pleuritic pain, arthritis, erysipelas-like exanthema, orchitis	Clinical criteria, MEFV gene
MKD	Recurrent fever, oral aphthous lesions, abdominal pain, diarrhea, vomiting, skin rash, generalized lymphadenopathy, splenomegaly, arthralgia, arthritis, myalgia	Episodes triggered by immunization at an early age
TRAPS	Non-regular and longer fever episodes, migratory skin rash, periorbital swelling, conjunctivitis, pleurisy, absence of vomiting and aphthous stomatitis	Autoinflammatory disorder with varied symptoms

FMF: Familial Mediterranean Fever, MKD: Mevalonate Kinase Deficiency, TRAPS: TNF-receptor associated periodic syndrome, ELANE Gene: Elastase Neutrophil Expressed.

**Table 3:** Gaslini score predicts the possibility of a genetic basis in patients presenting with PFAPA-like symptoms [10].

Gaslini Diagnostic Score	
Age at onset	
Positive family history	
Chest pain	
Abdominal pain	
Diarrhea	
Mouth ulcers	
Clinical symptoms grading	Never, sometimes, often, always
Score (>1.32)	High risk of hereditary forms of non-CAPS periodic fever syndromes
Sensitivity	>90% for proven gene mutation
Specificity	59–82%, significant number tested with no mutation found

CAPS: Cryopyrin-Associated Periodic Syndrome. Gaslini score can be automatically calculated in the site (https://www.printo.it/eurofever/periodic_fever, accessed on 24 March 2025).

**Table 4:** Past and present therapeutic options for PFAPA syndrome.

Currently Used Therapies				
Therapy	Evidence	Degree of Evidence	Effect	Comments
Corticosteroids	Various studies	High-IIB	Cessation of the attack	Commonly used to control inflammation
Anakinra	Clinical trials	Medium-IV	Decrease attack frequency	IL-1 receptor antagonist
Colchicine	Various studies	Medium	Decrease attack frequency	More efficient in MEFV (+) patients
Tonsillectomy	Clinical trials	High-IA	Decrease attack frequency	Not efficient in MEFV (+)
Past Therapies				
Therapy	Evidence	Degree of Evidence	Effect	Comments
Cimetidine	Various studies	Low	Decrease attack frequency	Histamine H2-receptor antagonist

MEFV: Mediterranean Fever gene, MEFV (+): Mediterranean Fever gene mutation carrier, IL-1: Interleukin-1.

**Table 5:** Experimental therapies.

Experimental Therapies				
Therapy	Evidence	Degree of Evidence	Effect	Comments
Lidomide	Anecdotal reports	Low	Decrease attack frequency	Potential immunomodulatory effects
Montelukast	Anecdotal reports	Low	Decrease attack frequency	Leukotriene receptor antagonist
Thalidomide		Medium	Decrease attack frequency	Adult PFAPA patients, reduction in oral ulcers and systemic symptoms
Vitamin D		Low	Decrease attack frequency	Thought to support immune function
Pidotimob		Low	Decrease attack frequency	Experimental therapy with potential benefits
Probiotic K12		Low	Decrease attack frequency	Probiotic strain with potential immune benefits

**Table 6:** Management based on CARRA consensus treatment plans [70].

PFAPA Management Steps	Description
(1) Antipyretic Arm	Symptomatic
(2) Corticosteroid Arm	Prednisolone 1 mg/kg initial single dose: <14 d interval between febrile episodes change to another arm 14–21 d interval trial of 2 mg/kg prednisolone and according to response: >21 d interval continue with that dose <21 d interval continue with another arm
(3) Prophylaxis Arm	Colchicine or Cimetidine
(4) Surgical Arm	Tonsillectomy

## Abbreviations

AIDA	AutoInflammatory Diseases Alliance
CAPS	Cryopyrin-Associated Periodic Syndrome
CARRA	Childhood Arthritis and Rheumatology Association
ELANE	Elastase Neutrophil Expressed gene
CARD8	Caspase recruitment domain-containing protein 8
FMF	Familial Mediterranean Fever
IgA	Immunoglobulin A
IL-1	Interleukin-1
IL-1β	Interleukin-1 beta
IL-1RN	Interleukin-1 receptor antagonist
MEFV	Mediterranean Fever
MKD/HIDS	Mevalonate Kinase Deficiency/HyperImmunoglobulin D Syndrome
NOD2	Nucleotide-binding oligomerization domain-containing protein 2
PFAPA	Periodic Fever, Aphthous Stomatitis, Pharyngitis, and Cervical Adenitis
PIK3AP1	Phosphoinositide-3-Kinase Adaptor Protein 1
RhoA	Member of the RhoA family
SAA	Serum Amyloid A
SPON2	Spondin 2
SURF	Undifferentiated Recurrent Fever Syndrome
Th1	T-helper cells 1
Th2	T-helper cells 2
TRAPS	TNF-receptor-associated periodic syndrome
